# Teaching away from school: do school digital support influence teachers’ well-being during Covid-19 emergency?

**DOI:** 10.1186/s40536-023-00159-7

**Published:** 2023-03-27

**Authors:** Melisa L. Diaz Lema, Lidia Rossi, Mara Soncin

**Affiliations:** School of Management-Politecnico Di Milano, Via Lambruschini 4/B, Milan, Italy

**Keywords:** Teachers’ well-being, School digital support, REDS data, School effect, Covid-19

## Abstract

The Covid-19 pandemic coerced the closure of most schools around the world and forced teachers and students to change teaching and learning methods. Emergency Remote Teaching (ERT) generated consequences to teachers and students in terms of learning outcomes and personal well-being. This study focuses on teachers’ individual and working environment well-being in ERT conditions and intends to explore which factors related to the provision of digital equipment and the implementation of digital strategies by schools explain the school effect on both typologies of well-being during the Covid-19 emergency. To do so, data collected in the Responses to Educational Disruption Survey (REDS) across three countries were used, and a two-step analysis was conducted. A first step involves the use of linear mixed effect models to assess the school effect on teachers individual and working environment well-being. In the second step Regression Trees (RT) are used to investigate which factors and policies related to digital tools explained the identified school effects. The results show that schools and countries played a role in determining teachers perceived well-being during the Covid-19 disruption, in particular the school level explains more than 7% of the work environment well-being and 8% of individual one. In the second step of the analysis results show that a high positive effect on school environment well-being is observed when the school’s activity is not influenced by policies limiting the use of online tools and when teacher’s readiness for remote teaching, like the development of technical skills and the provision of internet access and digital devices, is met. To the best of our knowledge, this is the first study that evaluates the impact of digital tactics and instruments provided by schools on teachers’ well-being on a large scale.

## Introduction

The Covid-19 pandemic has impacted the functioning of education systems in a multitude of ways by radically changing how teaching and learning happens to guarantee school continuity. During the pandemic peak almost the entire world population of students (more than 90 percent) suffered the closure of their school due to the policies undertaken to deal with the crisis (UNICEF, 2022). During this time, teachers, students, and families had to interact in new and completely remote ways, in a forced situation that has generated many drawbacks (Loukomies & Juuti, 2021).

Recent research has demonstrated the negative impact that school closure had on student performance (Engzell et al., 2021; Maldonado & De Witte, 2022) as well as on student well-being (Gaxiola Romero et al., 2022; Vira & Skoog, 2021). During the weeks of school closure, the role of families in supporting student remote schooling increased significantly (Gaxiola Romero et al., 2022), although the most prominent role has been played by teachers (Choi et al., 2021). Given that previous research stressed the link between teachers’ well-being and core measures like teaching effectiveness and student outcomes (Hascher & Waber, 2021), it is worth to investigate which factors are related to teachers’ well-being in the context of an Emergency Remote Teaching (ERT) (Portillo et al., 2020) like the one created by the Covid-19 crisis.

Scholars are increasingly investigating the factors related to students’ and teachers’ well-being during Covid-19, but usually under-investigate the function that schools may have in affecting this outcome. Indeed, given the ERT setting in which the school systems suddenly found to operate, school factors as digital devices, digital guidelines, and prompt indications about how to deliver ERT may affect the well-being of teachers and, in turn, impact on students cognitive and non-cognitive outcomes. The goal of this study is to investigate to what extent teachers’ perceived individual and work-related well-being had been affected by schools’ capability of providing digital support to deliver education during the Covid-19 crisis across different countries. At a first stage, this study estimates the school effect on the teacher’s perceived well-being, i.e., the school level residuals of teachers’ well-being net of teachers’ characteristics and school contextual factors. Thus, the school effect is defined as what remains unexplained in the variability of teachers’ well-being once that exogenous factors such as teachers and school characteristics are taken into account. Then, the school effect is interpreted by exploring whether the digital equipment supplied by the school and the changes introduced to face the crisis influence the school effect. Thus, in this second step, we use endogenous factors (i.e., choices made by the school on how to react to the crisis) to explain the school level variability in teachers’ well-being (i.e., the previously mentioned school effect on teachers’ well-being).

The data used in this study refer to the Responses to Educational Disruption Survey (REDS) collected by IEA (International Association for the Evaluation of Educational Achievement). The dataset includes information on how students, teachers, and schools in eleven countries^1^ were prepared for distance learning in times of Covid-19 school closure, as well as during the subsequent reopening phase. The data of the Russian Federation (RUS), United Arab Emirates (ARE), and Uzbekistan (UZB) meet the expectation in terms of representativeness for this study, which aims to evaluate the effect of digital strategies used by schools to compensate for their closure on teachers’ subjective well-being. Thus, the research addresses the following question:

• What factors related to the provision of digital equipment and the implementation of digital strategies by schools explain the school effect on teachers’ well-being during the Covid-19 emergency?

This study contributes to understanding which aspects related to schools’ digital support played an important role in influencing the school effect on teachers’ perceived well-being. In doing so, we properly disentangle the individual factors related to teachers’ well-being from the decisions taken and implemented by the school by means of a two-step procedure. Furthermore, the study allows comparing findings across countries and, to the best of our knowledge, this is the first study that evaluates the effect of digital support provided by schools on teachers’ perceived well-being on a large scale.

The remainder of the paper is as follows: “Reference literature” section presents the reference literature, and “Context and data” section reports the data and methodology used. “Methodology” section discusses the results, while “Results” section illustrates the implications and concludes.

### Reference literature

Studies addressing pupils and teachers’ well-being during Covid-19 are emergent and fast developing (Cece et al., 2022; McCluskey et al., 2021) and represent the first research stream tackled by the present study. On this aspect, recent evidence highlights that pupils’ learning experiences were significantly lower for remote learning compared to the classroom learning (Walters et. al, 2021). However, it is also known that levels of students and teachers’ engagement varied considerably across schools (Green, 2020) and that digital instruments and competences were the most critical resource to support the educational process throughout the schools’ closure (Portillo et al., 2020).

In the context of this study, teachers’ well-being is defined as “teachers’ responses to the cognitive, emotional, health and social conditions pertaining to their work and their profession” (Viac and Fraser (2020), p.18). Following the literature, we define teachers’ well-being as composed by an individual psychological and personal well-being, and a work related well-being (i.e., school well-being) that refers to how teachers individually perceive their well-being within the school context and depending on the procedures that the school has carried out to face the emergency (Hascher & Waber, 2021). Recent research on teachers’ well-being during the Covid-19 period highlights that teachers felt more stressed, perceived a higher workload and felt a sense of isolation that negatively impacted on their perceived well-being (Cece et al., 2022; Hascher & Waber, 2021). However, some protective factors have been also stressed, namely a high locus of control and self-efficacy (Truzoli et al., 2021). It is also worth mentioning that some studies highlight a positive impact on teachers’ well-being during the emergency period, as reported by Cece et al. (2022) for physical education teachers in Switzerland, who perceive on average higher collaboration, vigor scores and lower levels of physical fatigue. Given that the current study aims to contribute to the literature on school factors supporting teachers’ well-being rather than to the psychological literature, it is important to mention the findings coming from studies that stress the relevance of the availability of adequate digital tools and the perception of good digital competences on the well-being of teachers. In this strand, Lepp et al. (2021) qualitatively analyse teaching-related decisions during ERT by interviewing 16 Estonian teachers. The authors show that teaching decisions are mainly mediated by the availability of digital tools and by the ability (of teachers and students) to use them properly. Moreover, teachers tend to reduce the number of topics covered in order to safeguard their workload and well-being. Alves et al., (2021) conduct a survey among Portuguese teachers to assess the factors related to their well-being during the Covid-19 pandemic and show that the need to reinforce digital skills is a particularly relevant factor. Similar results have been highlighted by Carretero et al. (2021), who analyse the schooling practices during the Covid-19 pandemic in five EU countries (i.e., Belgium, Estonia, Greece, Italy and Poland) and show the relevance of good quality infrastructure and equipment as well as the need of reinforcing digital competencies to conduct quality teaching (during the emergency and beyond). Aligned to these findings are also the results by König et al. (2020) in the German context, resulting from a survey conducted among early-career teachers. The authors stress the relevance of teacher competence and school computer technology for positively facing the challenges posed by ERT. However, current evidence is focused on single-countries investigation, making it difficult to compare results at an international level. Moreover, large scale international datasets have not been explored so far, thus current evidence is based on small scale samples.

Furthermore, previous research focuses on teachers’ perceptions only, without disentangling digital school strategies/choices from the teachers’ perception of their usefulness and relative impact on wellbeing. The current research moves from these premises by taking advantage of the evidence on school effects as measured by multilevel models (Masci et al., 2016; Sellström & Bremberg, 2006). The current literature on school effects stresses the relevance of such measures of school effectiveness to support school accountability (Everson, 2017). The output measure of this kind of models is traditionally student achievement, thus the school effect indicates what the school adds to student achievement net of individual and contextual factors (Guarino et al., 2019; Li, Kennedy, & Mok, 2016). To do so, models account for the starting ability of students by using prior achievement as a control factor, and for this reason they are also called’value-added models’ (Everson, 2017; Li et al., 2016). The current research moves from the literature on school effects using teachers’ well-being as the output of interest, and applying multilevel models to estimate the school effects on teachers’ well-being. Despite the possibility to observe teachers only cross-sectionally, this approach allows disentangling the portion of variance in teachers’ well-being that is attributable to the school level, which allows studying the school effect from a new perspective.

## Context and data

### Research context

REDS data aim to capture a picture of national responses to the global pandemic. The goal of the data set is to document the effects of the Covid-19 pandemic on both school staff and students, while also gathering information on the various mitigation strategies that schools have implemented during this challenging period. REDS dataset is comprehensive of eleven countries in total: Burkina Faso, Denmark, Ethiopia, India, Kenya, the Russian Federation, Rwanda, Slovenia, the United Arab Emirates, Uruguay, and Uzbekistan. In these countries, school principals, students, and teachers of grade 8 were administered questionnaires that probed their experiences with the changes in schooling during the Covid-19 emergency (UNESCO, 2022). However, due to constrains on the comparability and representativeness of the data, our study focused on the teachers and schools’ questionnaires of only the Russian Federation (RUS), the United Arab Emirates (ARE), and Uzbekistan (UZB).

The three countries under investigation present similarities and differences in the reaction of the respective educational systems to Covid-19 emergency. To provide some contextual information, according to UNESCO (2022) and up to March 2022, ARE is the country with the longest school closure, lasting for nearly 18 weeks plus additional 47 weeks of partial opening. RUS and UZB are more similar in this respect, given that RUS had no complete school closure and 19 weeks of selective closure, while UZB had 13 weeks of complete closure and 1 week of selective closure. According to The World Bank (2022) the number of internet users by country is quite heterogeneous, given that it reaches the 100 percent of the population in ARE, while it represents the 85 percent in RUS and the 71 percent in UZB, the country with the lowest internet access among the three. This is reflected by the distance learning modalities adopted during school closure. Indeed, UZB is the only country in which education continuity was guaranteed not only through the internet but also through television programmes offered to students (Chicherina, 2020). Similar actions were also undertaken with respect to online/TV programmes offered to train teachers, support for school principals and agreements with internet providers for free connection (Chicherina, 2020; Erfurth & Ridge, 2020; Kosaretsky, Zair-Bek, Kersha, & Zvyagintsev, 2022). These countries characteristics will be helpful in the interpretation of the results.

### REDS questionnaires

The school questionnaire of REDS data collected information on school characteristics and school responses during the Covid-19 disruption period, including schools’ modifications to the teaching and learning arrangements. The questionnaire administered to teachers, besides each teacher’s background, focused on their response to the period of disruption, with respect to their teaching practices and their perceptions of the impact caused by the disruption on their well-being. From the selected countries, after managing missing data^2^ the final sample used in this study comprises in total 5789 teachers nested in 470 schools.

Teachers’ information related to well-being collected by the questionnaire intended to seize a comprehensive picture of the factors associated with individual well-being, but also what had been done within the school system to support the working environment well-being during the disruption period. In this regard, this study measures the school effect on well-being by aggregating in a comprehensive measure (the sum of all the components) REDS questionnaire items where teachers stated their perceived individual and working environment well-being (Table 1), controlling, at a teacher level, for background characteristics, living conditions during the disruption and working conditions. As shown in Table 1, the original scale of some of these items was transformed to a binary response to better grasp their impact. At a school level, this study first accounted for the contextual school factors, intended as non-manageable school characteristics like the percentage of students with low socio-economic status (SES) or the number of teachers in the school. REDS data for the selected sample show that during the Covid-19 emergency, teachers state to feel fatigue most of the time, feel isolated when working from home and have high concerns about catching Covid-19 at work. On average, they spend around four hours a day (about 240 min), teaching students remotely and spend much more of their time looking for new materials and activities, planning lessons and assessing students learning (Table 1). Information related to school capacity and resourcing support provided by the 470 schools are considered then to measure which digital initiatives and/or equipment supplied by schools to sustain ERT, like the provision of digital devices or Learning Management Systems, are eventually associated to the school effect on teachers’ perceived well-being during the disruption period (Table 2). As before, some of the questionnaire items are transformed to a binary response to capture their effect more in detail. Around 90% of the schools in the sample declare they have provided digital devices for some or all staff during the disruption as well as a Learning Management System. However, 70% of them state to have limited capacity due to the available technical skills for remote teaching, and half of them for having policies limiting the use of online tools.

**Table 1:** REDS questionnaire items used to measure school effect on perceived individual (Outcome 1) and working environment well-being (Outcome 2)

Level	Category	QRE-Item	Type	Description	Min	Mean	Max	SD	# obs
Teacher	Outcome 1: individual well-being	IT1G16F	Scale	Felt the need of assistance to support my well-being (1 = Strongly agree; 4 = Strongly disagree)	1	2,6	4	0,8	7.816
		IT1G16G^a^	Scale	Able to use my own methods (e.g. meditation) to cope with stress (1 = Strongly disagree; 4 = Strongly agree)	1	2	4	0,8	7.814
		IT1G16I^a^	Scale	Able to maintain my normal exercise and health routines (1 = Strongly disagree; 4 = Strongly agree)	1	2,1	4	0,8	7.828
		IT1G16J	Scale	Felt fatigued most of the time (1 = Strongly agree; 4 = Strongly disagree)	1	2,7	4	0,9	7.814
		IT1G16L	Scale	Sleeping patterns were interrupted (1 = Strongly agree; 4 = Strongly disagree)	1	2,6	4	0,9	7.822
	Outcome 2: Well-being in the in the working environment	IT1G16A	Scale	Concerns about catching Covid-19 at work (1 = Strongly agree; 4 = Strongly disagree)	1	3,2	4	0,9	7.845
		IT1G16B^a^	Scale	Satisfaction with the infection control protocols at my school (1 = Strongly disagree; 4 = Strongly agree)	1	1,6	4	0,7	7.832
		IT1G16D^a^	Scale	Able to cope with changes in teaching methods (1 = Strongly disagree; 4 = Strongly agree)	1	1,7	4	0,6	7.832
		IT1G16E^a^	Scale	Able to meet all the requirements of my job (1 = Strongly disagree; 4 = Strongly agree)	1	1,7	4	0,6	7.830
		IT1G16H^a^	Scale	I had time to interact socially with colleagues (1 = Strongly disagree; 4 = Strongly agree)	1	2,3	4	0,8	7.819
		IT1G16K^a^	Scale	Able to balance work and personal duties (1 = Strongly disagree; 4 = Strongly agree)	1	2,1	4	0,7	7.816
		IT1G16M	Scale	Felt isolated while working at home (1 = Strongly agree; 4 = Strongly disagree)	1	2,6	4	0,9	7.813
		IT1G16N^a^	Scale	Felt in control of home working environment (1 = Strongly disagree; 4 = Strongly agree)	1	2	4	0,7	7.812
	Backgroud	IT1G22	Binary	Gender (1 = Male)	0	0,3	1	0,4	8.068
		IT1G25	Scale	Teaching experience (1 = Less than 1 year; 6 = Over 20 years)	1	4,8	6	1,2	8.008
	Work characteristics	IT1G24A	Binary	Current employment status (1 = Part time)	0	0,2	1	0,4	7.943
		IT1G24B	Binary	Employment status during Covid-19 (1 = Part time)	0	0,1	1	0,3	7.376
		IT1G26A	Binary	Coordinator of a subject (1 = yes)	0	0,3	1	0,5	7.934
		IT1G26B	Binary	Coordinator of a no-teaching area (1 = yes)	0	0,1	1	0,3	7.869
		IT1G26C	Binary	Coordinator of a grade (1 = yes)	0	0,2	1	0,4	7.881
		IT1G26D	Binary	Coordinator of a sub-school (1 = yes)	0	0,1	1	0,3	7.863
		IT1G02^b^	Category	Teaching subject: (0 = humanities; 1 = math-science; 2 = other)	0	0,7	2	0,7	8.008
		IT1G01A	Scale	Minutes in a day teaching students face-to-face before Covid-19 (1 = less than 60 min; 7 = 360 min or more)	1	4,8	7	1,7	7.951
		IT1G11B^b^	Binary	Undertook professional learning using ICT in teaching (1 = yes)	0	0,9	1	0,3	7.932
		IT1G11C^b^	Binary	Undertook professional learning specific to the subject(s) you teach (1 = yes)	0	0,9	1	0,3	7.908
		IT1G11G^b^	Binary	Undertook professional learning in student well-being (1 = yes)	0	0,7	1	0,5	7.892
	Work (changing) conditions	IT1G03A^b^	Binary	Teaching remotely during Covid-19 (1 = yes)	0	0,4	1	0,5	7.993
		IT1G01B	Scale	Minutes in a day teaching students remotely during Covid-19 (1 = less than 60 min and 7 = 360 min or more)	1	4,7	7	1,8	7.922
		IT1G01C	Scale	Minutes in a day teaching students face-to-face during Covid-19 (1 = less than 60 min and 7 = 360 min or more)	1	2,9	7	2,2	7.875
		IT1G03BH^b^	Binary	Deployment of new activities in the online class (1 = yes)	0	0,8	1	0,4	7.367
		IT1G04H^b^	Binary	More time planning lessons during Covid-19 (1 = yes)	0	0,8	1	0,4	7.800
		IT1G05A^b^	Binary	Increase the time spent preparing lessons for the whole class (1 = yes)	0	0,8	1	0,4	7.839
		IT1G05B^b^	Binary	Increase the time spent managing student behaviour (1 = yes)	0	0,5	1	0,5	7.784
		IT1G05C^b^	Binary	Increase the time spent modifying work to suit students individual needs (1 = yes)	0	0,7	1	0,4	7.774
		IT1G05D^b^	Binary	Increase the time spent modifying teaching activities (1 = yes)	0	0,7	1	0,4	7.794
		IT1G05E^b^	Binary	Increase the time spent using materials provided by the school (1 = yes)	0	0,4	1	0,5	7.800
		IT1G05F^b^	Binary	Increase the time spent assisting students on a one-on-one basis (1 = yes)	0	0,7	1	0,4	7.810
		IT1G05G^b^	Binary	Increase the time spent looking for new teaching materials or activities (1 = yes)	0	0,9	1	0,3	7.806
		IT1G05I^b^	Binary	Increase the time spent assessing students learning (1 = yes)	0	0,7	1	0,5	7.808
		IT1G08A^b^	Binary	Increase student attendance (1 = yes)	0	0,2	1	0,4	7.866
		IT1G08C^b^	Binary	Increase student engagement during lessons (1 = yes)	0	0,2	1	0,4	7.829
		IT1G27B	Scale	Teaching time in subjects without qualifications during Covid-19 (1 = None; 5 = All my teaching time)	1	2	5	1,4	7.944
	Covid-19 living conditions	IT1G03CB^b^	Binary	Living with other adults (1 = yes)	0	0,6	1	0,5	7.351
		IT1G03CE^b^	Binary	Living with school-aged children at home (1 = yes)	0	0,5	1	0,5	7.349
		IT1G03CJ^b^	Binary	Balance between and home responsibilities (1 = Hard)	0	0,7	1	0,4	7.365
		IT1G18E^b^	Binary	Felt I needed to ask for professional support outside of my school (1 = yes)	0	0,4	1	0,5	7.911
		IT1G18G^b^	Binary	Felt supported by the local community (1 = yes)	0	0,6	1	0,5	7.902
School	School characteristics	IP1G31A^c^	Num	Percentage of girls in the school	0	50,4	100	15,1	494
		IP1G33A	Scale	Lowest grade taught in the school (1 = lower; 8 = highest)	1	1,4	8	1,2	507
		IP1G34	Scale	Population where the school is located (1 = less than 3,000 people and 5 = more than 1 million people)	1	3	5	1,3	508
		IP1G34A	Binary	Public or private (1 = Private)	0	0,2	1	0,4	508
		IP1G35A	Scale	Percentage of students whit a different first language (1 = less than 5\% and 5 = more than 50\%)	1	2,1	5	1,7	505
		IP1G35B	Scale	Percentage of students whit special needs (1 = less than 5\% and 5 = more than 50\%)	1	2,5	6	2,2	508
		IP1G35C	Scale	Percentage of students with low SES (1 = less than 5\% and 5 = more than 50\%)	1	1,6	5	0,9	505
		IP1G35D	Scale	Percentage of students with high SES (1 = less than 5\% and 5 = more than 50\%)	1	3,6	5	1,5	506
		IP1G35E	Scale	Percentage of students with an immigrant background (1 = less than 5\% and 5 = more than 50\%)	1	1,7	5	1,4	499
		IP1G35F	Scale	Percentage of students with a single-parent household (1 = less than 5\% and 5 = more than 50\%)	1	2	5	1	504
		IP1G36	Scale	Average class size before Covid-19 (1 = less or equal than 15 students and 6 = more than 35 students)	1	4,1	6	1,3	507
		IP1G37A	Num	Number of teachers	10	82,4	462	63,2	504
		IP1G37B	Num	Percentage of full-time teachers	0	81,9	100	31,3	499

^a^Original scale was inverted

^b^Original variable was re-scaled to a binary variable (1 = yes; 0 = no)

^c^Original value was transformed to relative terms

**Table 2:** REDS questionnaire items on school capacity and resourcing support used to explain the well-being school effect

Level	Category	QRE-item	Type	Description	Min	Mean	Max	SD	# obs
School	School capacity (SC) and resourcing support	IP1G02K^a^	Binary	SC limited during Covid-19 for lack of teachers (1 = yes)	0	0,2	1	0,4	505
		IP1G02L^a^	Binary	SC limited during Covid-19 for lack of experience with remote learning pedagogy (1 = yes)	0	0,6	1	0,5	505
		IP1G02M^a^	Binary	SC limited during Covid-19 for lack of technical skills to manage remote teaching (1 = yes)	0	0,7	1	0,5	504
		IP1G02Q^a^	Binary	SC limited during Covid-19 for policies limiting the use of online tools (1 = yes)	0	0,5	1	0,5	502
		IP1G03A^a^	Binary	Provided internet access for some or all staff (1 = yes)	0	0,8	1	0,4	511
		IP1G03B^a^	Binary	Provided internet access for some or all students (1 = yes)	0	0,7	1	0,5	510
		IP1G03C^a^	Binary	Provided digital devices for some or all staff (1 = yes)	0	0,9	1	0,3	509
		IP1G03D^a^	Binary	Provided digital devices for some or all students (1 = yes)	0	0,7	1	0,4	508
		IP1G03E^a^	Binary	Provided a Learning Management System (1 = yes)	0	0,9	1	0,3	509
		IP1G12C^a^	Binary	Changes in the provision of professional development activities for remote teaching (1 = yes)	0	0,8	1	0,4	508
		IP1G12E^a^	Binary	Changes in the provision of peer collaboration opportunities (1 = yes)	0	0,8	1	0,4	507
		IP1G17C^a^	Binary	Changes in the provision of support services for parents (1 = yes)	0	0,8	1	0,4	506

^a^Original variable was re-scaled to a binary variable (1 = yes; 0 = no)

## Methodology

### Measuring the school effect on teachers’ perceived well-being

In the first step of analysis, this study measures a construct of the self-perceived well-being of teachers, calculated using REDS questions as described in Table 1. From a methodological point of view, mixed effects models are used in this phase, using school and country as random effects and personal characteristics as fixed effects. Two set of models are constructed: one measuring the school effect on the perceived teachers individual well-being and other estimating the school effect to the perceived school environment well-being. Mixed effects modelling allows measuring the influence of the school on teachers’ perceived well-being, extracting the school effect, defined as the difference between the well-being value of a teacher in a school and the average value observed in schools populated by teachers with similar observable characteristics.

Very often observational data collected for the social sciences, and more particularly in the educational field, have a hierarchical or nested structure. Hierarchy is understood as units grouped at different levels: in the current study teachers nested in schools, in turn nested in countries. The existence of these hierarchies of data is neither accidental nor negligible: both the group and its members influence and are influenced by group membership (Goldstein, 2011).

Mixed effects models are exactly models designed to handle hierarchical data. The advantages of this type of modelling are many. First, it allows obtaining statistically efficient estimates of regression coefficients. Moreover, by using the clustering information it allows providing standard errors, confidence intervals and correcting significance tests. Third, it is possible to investigate the extent to which differences in results between schools are due to factors related to higher levels or other teachers’ characteristics since it is permissible to use covariates measured at any level of a hierarchy.

The models proposed for the empirical analysis have three levels in which teachers (level 1) are nested in schools (level 2), that are nested in countries (level 3). The model, for teacher $i, i=1,\dots , {n}_{lj};n=\sum_{lj}{n}_{lj}$, in school $l, l=1, \dots , {L}_{j};L=\sum_{j}{L}_{j},$ in country $j, j=1, \dots , J$ can be written as:

$${y}_{ilj}={\beta }_{0}+\sum_{k=1}^{K}{\beta }_{k}{x}_{kilj}+{b}_{j}+{u}_{lj}+{\varepsilon }_{ilj}$$

With $${b}_{j}\sim N\left(0; {\sigma }_{country}^{2}\right), {u}_{lj}\sim N\left(0; {\sigma }_{school}^{2}\right), {\varepsilon }_{ilj}\sim N\left(0; {\sigma }_{\varepsilon }^{2}\right)$$

Where ${y}_{ilj}$ is the measure of teachers’ perceived well-being *i*, in school *l*, in country *j*; *β* = *β*_0_*,…,β*_*K*_ is the (*K* + 1) − dimensional vector of parameters; ${x}_{kilj}$ is the value of the *k* − th predictor at teacher’s level; *b*_*j*_ is the random effect of country *j*; *u*_*lj*_ is the random effect of the school *l*, in country *j*; ${\varepsilon }_{ilj}$ is the error; and it is assumed that *b* is independent of $\varepsilon$ and *u* independent of $\varepsilon .$ (Masci et al., 2016). As a robustness check of the school effect estimation, single models for each country with two levels were also considered.

When using survey-collected data, it is generally important to incorporate weights into the analysis so that robust population estimates can be obtained (Goldstein, 2011). In particular, the unequal selection probabilities of the sampling units of REDS data necessitate the use of weights during computation of estimates. For this reason, weights variables are considered in the models using the package R WeMix by Bailey et al. (2020) for the implementation.

### The role of digital instruments to explain the school effect on teachers’ well-being

At this point, after extracting the school effect on teachers’ well-being from the three-levels models^3^ the study examines to what extent the provision of digital equipment and the implementation of digital strategies by the school is associated to the well-being school effect. In general, it intends to investigate what characteristics of schools or policies applied by schools determine a certain school effect increase or decrease on teachers’ perceived well-being. The variables used in this step are those described in Table 2.

In this second step of the analysis Regression Trees are applied (Breiman et al., 2017). Regression Trees (RT) are the development of ordinary Decision Trees used for numeric prediction (James, Witten, Hastie, & Tibshirani, 2013). RT follows a top-greedy algorithm that divides the covariates space into subspaces. The prediction is then computed as the average of the target numerical variable within the subspaces. (Witten, Frank, Hall, & Pal, 2016).

Decision Tree construction is a recursive procedure involving (i) the selection of the best splitting attribute and thus the selection of an adequate purity measure and (ii) pruning to avoid overfitting. In each node a portion *D* of the training data arrives and a splitting criterion is used to determine which attribute is the best to split it. In Regression Trees the splitting criteria aims at minimising the intrasubset variation in the class values down each branch. The standard deviation of the class values in *D* is treated as a measure of the error, so in each node the expected reduction in error for each attribute is computed. The attribute that maximises the expected error reduction is selected for splitting at the node. The SDR (standard deviation reduction) is defined as:

$$SDR= \sigma \left(D\right)- \sum_{i}\frac{\left|{D}_{i}\right|}{\left|D\right|}\sigma \left({D}_{i}\right)$$

where *D*_1_*,D*_2_*,…* are the sets that result from splitting the node according to the chosen attribute, and *σ*(*D*) is the standard deviation of the class values. The splitting process stops when the standard deviation of class values of the instances that reach a node is a small fraction of the standard deviation of the original instance set or when just a few instances remain. Once the basic tree has been formed, consideration is given to pruning the tree back from each leaf. To do that, an estimate of the expected error for the test data is computed for each node and they are dropped one by one as long as the error estimate decreases.

At this step, the advantages of using Regression Trees are multiple. From a more technical point of view, Regression Trees are useful since they allow relaxing the assumption of linearity; moreover, they do not require normalisation and scaling of data. On the practical side, they are easy to interpret, understand, and visualise.

## Results

### School effect on teachers perceived well-being

Schools and countries played a role determining teachers’ perceived well-being during the Covid-19 disruption. Tables 3 and 4 show the values of the proportion of unexplained variance by random effects (PVRE) considering two (when the analysis is done by country) and three level (multiple countries) models. In the latter ones, where all the countries are included, the school level explains 8.4% of the variability in the model for predicting individual well-being and about 7.2% in the model that predicts school well-being; the next level of hierarchy (i.e., country of origin) explains 6.1% and 2.4% of the variability, respectively^4^ As observed, the proportion of variance explained within countries (the school effect) is in both cases larger than the proportion of variance explained across countries (the country-effect), indicating a prevalent role of the institutional decisions at the school level on teachers’ perceived well-being during the educational disruption. In two of the three countries under analysis (i.e. RUS and ARE) the individual well-being is more sensitive to school and countries mechanisms, with respect to the school environment well-being. In all three cases, however, the largest proportion of variance explaining both typologies of well-being (i.e. the residual variance) is attributable at an individual level.

**Table 3:** PVRE by country and by school in teachers’ individual well-being

*Individual well-being*	School variance	school effect (%)	Country variance	Country-effect	Residual variance
All countries	0.391	8.4	0.283	6.1%	4.003
RUS	0.375	8.9	–	–	3.836
ARE	0.328	6.5	–	–	4.731
UZB	0.201	5.0	–	–	3.840

**Table 4:** PVRE by country and by school in teachers’ school-environment well-being

*School environment well-being*	School variance	School effect (%)	Country variance	Country-effect	Residual variance
All countries	0.550	7.2	0.186	2.4%	6.881
RUS	0.4139	5.5	–	–	7.1046
ARE	0.4208	5.9	–	–	6.673
UZB	0.4665	7.8	–	–	5.517

**Fig. 1:**
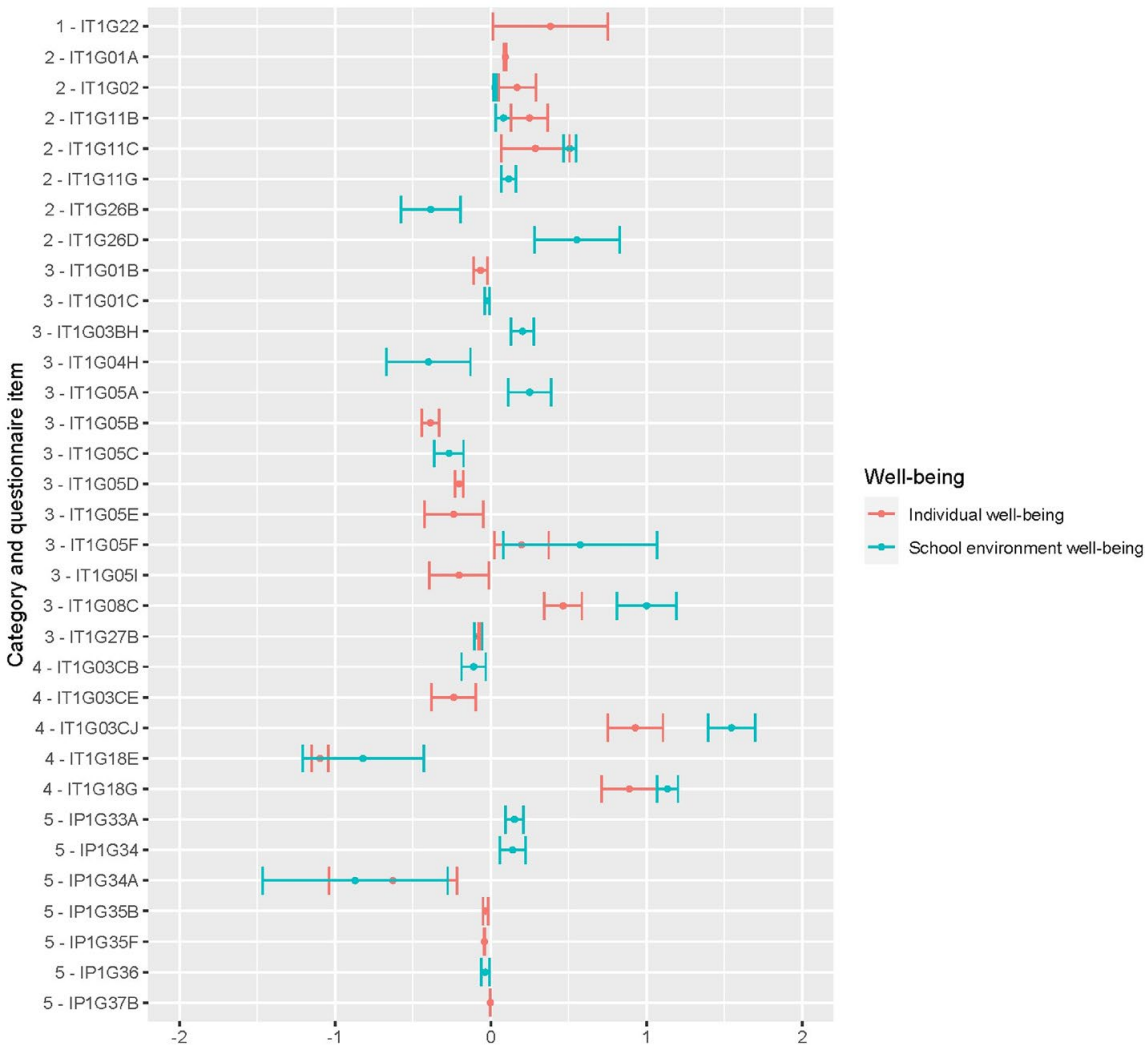
Coefficients estimates of Mixed Effect Models. Categories: 01-Background, 02-Working characteristics, 03-Work (changing) conditions, 04-Covid-19 living conditions, 05-School characteristics

To deepen the results, Fig. 1 shows the confidence intervals estimated from the significant coefficients, extracted from the mixed-effects models applied in the first step. Concerning background characteristics, being a male teacher (IT1G22) shows a positive effect on individual well-being, while teachers’ experience does not affect any type of teachers’ perceived well-being in a significant way. Spending a lot of time in face-to-face teaching before Covid-19 (IT1G01A) had a beneficial impact on individual well-being while engaging in professional learning (IT1G11B, IT1G11C) has a beneficial impact also in school-environment well-being. Some job responsibilities played a significant role with regards to individual well-being, being a coordinator of a non-teaching area (IT1G26B) had a detrimental impact on school well-being, while being a coordinator of a sub-school (IT1G26D) had a positive effect on it.

In terms of work-changing conditions, spending more time planning lessons (IT1G04H) and managing or modifying work to suit students’ needs during Covid-19 (IT1G05B, IT1G05C, IT1G05D, IT1G05E) had a negative impact, especially with regards to individual well-being, but spending time assisting students on a one-on-one basis (IT1G05F) and perceiving greater students’ engagement (IT1G08C) during lessons were beneficial for teachers in both typologies of well-being.

Regarding teachers’ living conditions, being able to manage a balance between work and home responsibilities and feeling supported by the local community (IT1G03CJ, IT1G18G) are associated to a large positive impact on well-being, while teachers feeling the need for professional support (IT1G18E) report a detrimental effect on well-being during the disruption period. Referring to the impact of school characteristics on well-being, teachers working in a private school (IT1G34A, 20% of our sample) are associated with a lower level of well-being in both categories, while teachers working in higher school orders (IP1G33A), and in schools located in a populated area (IP1G34) had a positive effect on perceived individual well-being during the disruption.

### Digital instruments explaining the school effect on teachers’ perceived well-being by country

**Fig. 2:**
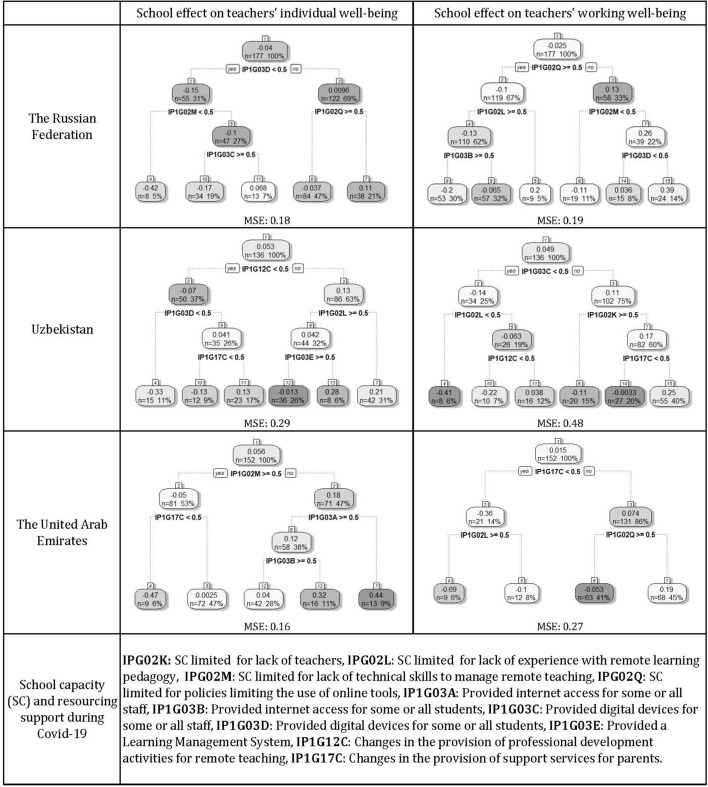
Digital instruments and strategies associated wit school effect on teachers perceived well-being by country

In order to explore the heterogeneity behind the school factors that can help to explain the school effect on teachers well-being, the following section presents the second step of the analysis by country. The trees obtained as output in this phase of the analysis are shown in Fig. 2, which also reports the respective Mean Squared Error (MSE) to compare predictive accuracy across countries. The baseline results are confirmed, despite the relevant differences characterising each case.

The contextual factors presented in “Research context” section support the explanation of the results, which highlight some commonalities and differences across countries. In the Russian context, the factors bringing to the most positive school effect on school environment well-being is observed when the school’s activity is not influenced by policies limiting the use of online tools (e.g., limitations on how teacher can interact with students online, IP1G02Q), when teachers have sufficient technical skills (IPG02M) and when digital devices are provided to students (IP1G03D). These findings show the importance of teachers’ room for freely organise teaching activities, teachers’ skills, and students’ digital equipment (as an enabling factor). When looking at school effect on individual well-being, the provision of digital devices to students (IP1G03D) emerges again as a fundamental factor for positive school effect, together with the absence of restrictive policies (IP1G02Q). Thus, the main difference is represented by the factor related to teachers’ technical skills that mainly influences the school environment, and probably the regular functioning of teaching activities.

Results referred to ARE show, as the first splitting factor, the changes in the provision of support services for parents (IP1G17C) as well as the absence of policies limiting the use of online tools (IP1G02Q), similarly to RUS. When looking at the school effect on individual well-being, factors related to teachers’ readiness emerge, in particular the possession of technical skills (IPG02M) and the provision of internet access to the staff (IP1G03A) are associated to the highest school effect. This is in line with the idea that the factors that influence the school effect on individual well-being are more closely related to teachers’ readiness to face remote learning.

Finally, results from UZB show that the factors most positively affecting school effect on school environment well-being are the provision of digital devices to the staff (IP1G03C), an adequate presence of teachers to run school activities (IPG02K) and the changes in the provision of support services for parents (IP1G17C). On the other hand, the school effect of individual well-being is positively affected by the provision of professional development activities (IP1G12C) and by the possession of adequate experience with remote learning pedagogy (IPG02L), but without the provision of a Learning Management System (LMS) by the school (IP1G03E). It might be the case that in the Uzbek context, in which the use of online remote teaching is more limited and teaching activities were often carried out on Telegram through homework assignment and correction (Chicherina, 2020), the country benefited the most from having enough well-trained teachers on remote teaching pedagogy, while the presence of a LMS is not crucial in this respect.

## Conclusions

This study shows that schools had a relevant role on teacher’s perceived well-being in the context of ERT, and digital tactics and instruments adopted by the schools across countries did have an incidence on the well-being school effect.

This research contributes to the literature on school effects using teachers’ well-being as the output of concern, disentangling the portion of variance in teachers’ well-being that is attributable to both school and country levels.

Aligned with previous literature, this study finds, at a large scale, that digital skills (Alves et al., 2021) and quality infrastructure and equipment (Carretero et al., 2021) provided by the school are relevant factors related to teachers’ well-being to face the challenges resulted from ERT. Yet, in the country with less technical infrastructures (UZB), pedagogical techniques and professional development activities for remote teaching are perceived as more important than technical skills. This may be related to the instruments used to carry out ERT. 84% of UZB teachers interacted with students during the disruption period mainly through Telegram (Chicherina, 2020), a communication platform whose technical requirements are popularly known for its daily life usage. Nonetheless, the IT divergence in UZB also shows how Learning Management Systems have an important role on the school effect related to teachers’ working environment well-being.

On the other hand, countries with a larger IT coverage such as RUS and ARE show that factors that influence the school effect on individual well-being referred to teachers’ readiness to face remote learning, like internet access and the possession of technical skills. However, teachers’ and schools’ entitlement of using online tools for remote teaching without major limitations is also similarly important.

The only factor that did not play a significant part on the well-being school effect in any of the evaluated scenarios during the disruption is the increase in peer collaboration opportunities, even though the time dedicated to it during the Covid-19 disruption increased significantly.

This study allows comparing findings across countries on teachers’ well-being school effect and, to the best of our knowledge, this is the first study that evaluates the impact of digital tactics and instruments provided by schools on teachers’ well-being on a large scale. However, the data are based on a cross section of observations collected in a single point in time. Further research could explore in greater detail how the well-being of teachers and the school strategies have evolved after the emergency period. The results could also be enriched by observing student outcomes, that could provide a more complete picture of the school effects generated. Finally, having a larger set of countries would enhance findings comparability.

## Data Availability

The datasets generated and/or analysed during the current study are available in the REDS data repository, https://www.iea.nl/data-tools/repository/reds.

## Annex

We found a strong correlation between the well-being school effect computed with the additive composite indicator used in the paper, and an additional well-being indicator computed through Factor analysis.

- Individual well-being: r(468) = 0.91, p < 2.2e-16.
- School-environment well-being: r(468) = 0.79, p < 2.2e-16.

The proportion of unexplained variance by random effects (PVRE) and school effects using a Factor analysis to compute the composite well-being indicators are shown in the Table 5.

**Table 5:** PVRE by country and by school in teachers’ well-being

*Teachers’ well-being*	School variance	School effect (%)	Country variance	Country-effect (%)	Residual variance
Individual	0.062	9.2	0.032	4.7	0.578
School-environment	0.035	4.3	0.001	0.2	0.767

## Footnotes

1 All eleven REDS participants are: Burkina Faso; Denmark; Ethiopia; India; Kenya; Russian Federation; Rwanda; Slovenia; United Arab Emirates; Uruguay; Uzbekistan.

2 As far as independent variables are concerned, observations with NA in some of them were removed; alternatively, when an NA is found in only one of the variable used to construct the dependent variables of individual or school environment well-being, they were imputed as average value of the other responses.

3 The school effects estimated with the two and the three-level models are highly correlated, with regard to individual well-being, the correlation test indicates r(468) = .89, p < 2.2e-16, while for the school environment well-being is r(468) = 0.83, p < 2.2e-16.

4 The school effects estimated are consistent even considering an alternative aggregated measure of well-being as reported on the Annex 1.
